# The complete mitochondrial genome of *Tetramorium tsushimae* (Emery, 1925) (Hymenoptera: Formicidae)

**DOI:** 10.1080/23802359.2021.2008830

**Published:** 2021-12-10

**Authors:** Ru-Yi Yin, Yi Luo, Zhao-Min Zhou

**Affiliations:** a Key Laboratory of Southwest China Wildlife Resources Conservation (Ministry of Education), China West Normal University, Nanchong, China; b Key Laboratory of Environmental Science and Biodiversity Conservation (Sichuan Province), China West Normal University, Nanchong, China

**Keywords:** *Tetramorium tsushimae*, Myrmicinae, mitochondrial genome, phylogenetic analysis

## Abstract

*Tetramorium tsushimae* (Emery, 1925) is an omnivorous ant species native to East Asia and has been introduced to North America. The mitochondrial genome of *T. tsushimae* is 19,207 bp in length with an A + T content of 81.3% and includes 13 protein-coding genes, 2 ribosomal RNA genes, 22 transfer RNAs, and a control region. This sequence data would play an important role in the investigation of mitochondrial evolution of the subfamily Myrmicinae.

*Tetramorium tsushimae*, the Japanese pavement ant, usually occurs in urban habitats with open, sunny, bare or grassy land (Sanada-Morimura et al. 2006), which is an omnivorous species with seed-carrying behavior participating in plant distribution (Ohtsuka et al. 2020), predation activities shifting social organization of the halictine bee (*Lasioglossum baleicum*) (Yagi and Hasegawa 2011) and attending effects on an aphid (*Macrosiphoniella yomogicola*) (Watanabe et al. 2019). This species was introduced to North America from the Eastern Palearctic probably in the first half of the 20th century (Steiner et al. 2006). As one of the species-rich myrmicine genera, *Tetramorium* constitutes Crematogastrini group with *Cardiocondyla*, *Crematogaster*, *Pristomyrmex*, *Vollenhovia* and about 50 other genera, while relationships were not well resolved at the base of this group based on nuclear gene fragments, likely due to a period of rapid diversification early in its history (Ward et al. 2015). Therefore, to better understand the phylogenetic relationship, we present the complete mitochondrial genome of *T. tsushimae* as the first mitogenome of the genus *Tetramorium*.

Worker ants of *T. tsushimae* used in this study were collected in Nanchong City (30°48′57.43″N, 106°3′36.33″E), China in October 2020. The collected specimens of this species were deposited at the Key Laboratory of Southwest China Wildlife Resources Conservation, China West Normal University (www.cwnu.edu.cn; contact Yi LUO, v_luoyi@126.com) after morphological identification, under the voucher number NCTT202010. Total genomic DNA was extracted and sequenced on the Illumina Novaseq sequencing platform by Shanghai Personal Biotechnology Co. Ltd, China. High-quality reads were then assembled de novo using A5-miseq v20150522 (https://github.com/koadman/docker-A5-miseq) (Coil et al. 2015) and SPAdes v3.9.0 (http://cab.spbu.ru/software/spades/) (Bankevich et al. 2012). Mitogenome annotation was conducted with the MITOS Web Server (Bernt et al. 2013). Finally, we submitted the complete mitochondrial genome sequence to GenBank and was given an accession number MW429350.

The mitochondrial genome of *T. tsushimae* is 19,207 bp long and contains 38 genes including 13 protein-coding genes (PCGs), 2 rRNA genes, 22 tRNA genes and a control region. The nucleotide composition is AT-biased (81.3%). Four PCGs (*nad4*, *nad4l*, *nad5*, and *nad1*) are encoded by the majority strand (J-strand) while the other nine are located on the minority strand (N-strand). All PCGs use ATN as the start codons (seven ATT, five ATG, one ATA) and TAA as the stop codon. The tRNAs size varies from 58 bp (*trnS1*) to 70 bp (*trnR*). The lengths of *rrnL* and *rrnS* are 1265 and 720 bp, with AT contents of 82.1 and 84.2%, respectively. The control region is 1167 bp long with a high AT content of 93.8%. The gene order (GO) of *T. tsushimae* has three rearrangements compared to the ancestral insect (Cameron 2014) (ancestor GO: *T. tsushimae* GO; *trnR*- *trnN*- *trnS1*: *trnN*- *trnR*- *trnS1*-; *rrnL*- *trnV*- *rrnS*: *rrnL*- *rrnS*- *trnV*; *trnI* -*trnQ*- *trnM*: *trnM*- *trnI* -*trnQ*), with a common future of Myrmicinae ants that 12S rRNA is located before *trnV* (Babbucci et al. 2014; Park et al. 2020).

We selected 28 ants and an outgroup species, *Apis mellifera ligustica*, to infer the phylogenetic relationships. Thirteen PCGs and two rRNA genes were aligned using MAFFT (Katoh and Standley 2013) plugin in PhyloSuite v1.2.2 (Zhang et al. 2020), respectively. The best-fit model was GTR + F + I + G4, selected by ModelFinder (Kalyaanamoorthy et al. 2017), and the Bayesian inference (1,100,000 generations, sampled every 100 generations) tree was constructed using MrBayes method by PhyloSuite. The maximum likelihood and neighbor-joining trees were constructed using MEGA X (Kumar et al. 2018) with 10,000 bootstrap replicates. Although the phylogenetic tree showed the *T. tsushimae* is most closely related to *Cardiocondyla* and *Crematogaster* (Figure 1), the monophyly of Crematogastrini remains uncertain, because *Pristomyrmex* and *Vollenhovia* belong to other clades with weak supports. This uncertainty is congruent with the previous mitogenome research (Park et al. 2020). *T. tsushimae* mitogenome will aid our understanding of the mitochondrial evolution of ants.

**Figure 1.**
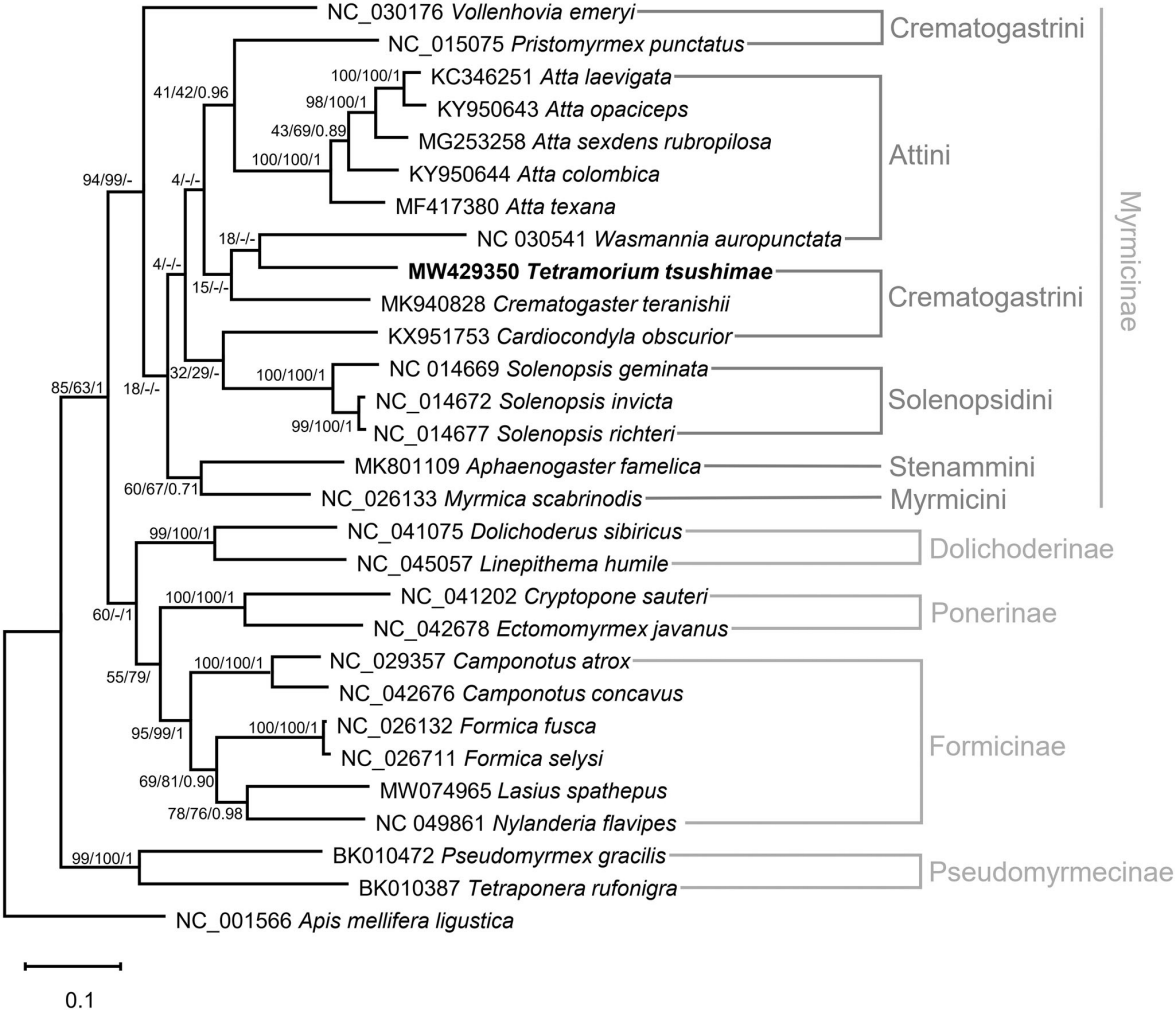
Maximum likelihood, neighbor-joining, and Bayesian inference phylogenetic trees based on the concatenated PCGs and two rRNA genes of 29 Hymenoptera species (28 ants and one bee). Phylogenetic tree was drawn based on the maximum likelihood phylogenetic tree. Numbers at nodes indicate bootstrap support values of maximum likelihood and neighbor-joining trees, and posterior probability of Bayesian inference tree, respectively.

## Data Availability

The data that support the findings of this study are openly available in GenBank of NCBI at https://www.ncbi.nlm.nih.gov, reference number MW429350. The associated BioProject, SRA, and Bio-Sample numbers are PRJNA772530, SUB10545189, and SAMN22374998, respectively.
